# Mud Ring Optimization Algorithm with Deep Learning Model for Disease Diagnosis on ECG Monitoring System

**DOI:** 10.3390/s23156675

**Published:** 2023-07-26

**Authors:** Ala Saleh Alluhaidan, Mashael Maashi, Munya A. Arasi, Ahmed S. Salama, Mohammed Assiri, Amani A. Alneil

**Affiliations:** 1 Department of Information Systems, College of Computer and Information Sciences, Princess Nourah Bint Abdulrahman University, P.O. Box 84428, Riyadh 11671, Saudi Arabia; 2 Department of Software Engineering, College of Computer and Information Sciences, King Saud University, P.O. Box 103786, Riyadh 11543, Saudi Arabia; 3 Department of Computer Science, College of Science and Arts in RijalAlmaa, King Khalid University, Abha 62529, Saudi Arabia; 4 Department of Electrical Engineering, Faculty of Engineering & Technology, Future University in Egypt, New Cairo 11845, Egypt; 5 Department of Computer Science, College of Sciences and Humanities-Aflaj, Prince Sattam bin Abdulaziz University, Aflaj 16273, Saudi Arabia; 6 Department of Computer and Self Development, Preparatory Year Deanship, Prince Sattam bin Abdulaziz University, Al-Kharj 16278, Saudi Arabia

**Keywords:** ECG signals, cardiovascular disease, deep learning, hyperparameter tuning

## Abstract

Due to the tremendous growth of the Internet of Things (IoT), sensing technologies, and wearables, the quality of medical services has been enhanced, and it has shifted from standard medical-based health services to real time. Commonly, the sensors can be combined as numerous clinical devices to store the biosignals generated by the physiological actions of the human body. Meanwhile, a familiar method with a noninvasive and rapid biomedical electrocardiogram (ECG) signal can be used to diagnose and examine cardiovascular disease (CVD). As the growing number of patients is destroying the classification outcome because of major changes in the ECG signal patterns among numerous patients, computer-assisted automatic diagnostic tools are needed for ECG signal classification. Therefore, this study presents a mud ring optimization technique with a deep learning-based ECG signal classification (MROA-DLECGSC) technique. The presented MROA-DLECGSC approach recognizes the presence of heart disease using ECG signals. To accomplish this, the MROA-DLECGSC technique initially preprocessed the ECG signals to transform them into a uniform format. In addition, the Stacked Autoencoder Topographic Map (SAETM) approach was utilized for the classification of ECG signals to identify the presence of CVDs. Finally, the MROA was applied as a hyperparameter optimizer, which assisted in accomplishing enhanced performance. The experimental outcomes of the MROA-DLECGSC algorithm were tested on the benchmark database, and the results show the better performance of the MROA-DLECGSC methodology compared to other recent algorithms.

## 1. Introduction

The Internet of Things (IoT) has attracted much attention in industries and academia. The IoT can be considered as an expansion of the Web application that is the Internet connection with real-life things apart from data [1]. It is an indispensable part of the new generation of information technology that can be broadly utilized in the fusion of the network using communication technologies, namely pervasive computing, smart perception [2], and recognition technology. Simultaneously, the use of the IoT in medical treatment is gaining more popularity, leading to smart medical care, for example, digitization of therapeutic processes, visualization of material management, and digitization of medical information [3]. Smart healthcare is a technology that brings about better treatment for patients, better diagnostic devices, and tools that can enhance people’s standard of living [4]. In this study, smart healthcare regarding cardiovascular diseases is much more focused. Heart or cardiovascular disease is most of the life-threatening diseases that cause higher mortality rates all over the world [5]. Annually, the number of heart disease cases is estimated to be 17.7 million, which is nearly 31% of the global casualties. Few cardiovascular diseases are undetectable and asymptomatic, namely atrial fibrillation, that has to monitor ambulatory electrocardiography in the long term [6]. Electrocardiograms (ECGs) can be referred to as a type of diagram generated from the heart’s electrical activities in terms of time and voltage. Certain monitoring tools can record it [7].

As everyone knows, deep learning (DL) is a subdivision of machine learning (ML) that learns complicated representations of data by using many layers of interconnected neurons. By examining large quantities of medical datasets, like medical images or patient records [8], DL methods can learn to find patterns that are related to particular diseases, like heart disease or cancer. DL methods were utilized for identifying patterns and examining patient records that are linked with different diseases [9]. By examining great quantities of patient data, like demographic information, medical histories, and lab results, DL methods can learn to find risk factors for numerous diseases and make predictions regarding the possibility of a patient developing a specific disease [10].

This study presents a mud ring optimization method with a deep learning-based ECG signal classification (MROA-DLECGSC) algorithm. The MROA-DLECGSC technique initially preprocesses the ECG signals to transform them into a uniform format. In addition, the Stacked Autoencoder Topographic Map (SAETM) approach was utilized for the classification of ECG signals to identify the presence of CVDs. Finally, the MROA was applied as a hyperparameter optimizer, which assisted in accomplishing enhanced performance. The experimental outcomes of the MROA-DLECGSC technique were tested on the benchmark ECG signal dataset.

## 2. Related Works

Kumar et al. [11] modeled a DL- and fuzzy clustering-related method for arrhythmia identification from ECG signals. The augmented imageries were sent through CNN feature extractors. For classifying the ECG signals for heart diseases, the mined features were sent to a fuzzy clustering approach. In [12], to diagnose many cardiovascular diseases (CVDs) and for constant observing, a homecare-based ECG analysis platform was devised depending on large-scale, multilabel DCNN. In the meantime, targeting wearable applications or lightweight homecare, an algorithm–hardware co-optimizer was carried out to hasten the computation model over an embedding platform, including a field-programmable gate array (FPGA) for constant monitoring. The authors in [13] examined an automated classification and detection of arrhythmia with the projected and optimized DL algorithm. With the goal of making the QRS complex, the ECG signals gathered through the IoT nodes were processed, and the RR interval for accomplishing the feature vector that can be performed through the presented Coy–GWO-based deep CNN was abbreviated as Coy–Grey Wolf optimization-based DCNN technique that finds the anomalies in ECG signals.

Khanna et al. [14] presented a novel DL- and IoT-assisted clinical disease diagnosis (IoTDL-HDD) technique through biomedical ECG signals. Further, the presented IoTDL-HDD method used a BiLSTM extraction feature method for extracting valuable feature vectors in ECG signals. The artificial flora optimizer (AFO) method was used as a hyperparameter optimizer to enhance the efficacy of the BiLSTM method. In addition, a fuzzy DNN (FDNN) technique was leveraged to assign appropriate class labels to ECG signals. The authors in [15] modeled a possible cybertwin-related, multimodal network for ECG patterns observing day-to-day action. This network model has several cybertwin communication ends and a cloud-centric network. To enhance identification accuracy, the authors devised a new DCNN-HAR (human activity recognition).

In [16], the authors presented the IoT-oriented ECG monitoring structure with secure data broadcast for constant cardiac health observation. The ECG signals in the Physio Net Challenge databases and the MIT-BIH and ECG signals for different physical actions were checked and examined in real time. Suhail and Razak [17] modeled a structure for the automatic recognition of cardiovascular disease. The primary target is to design a method for future analysis of heart disease through symptom-based detection and ECG analysis. Nonlinear Vector Decomposed NN techniques and DWT were utilized for forecasting heart disease. The NN was inputted with 13 clinical features that can be trained later through a nonlinear vector decomposition of the absence or presence of cardiovascular diseases.

## 3. Proposed Model

In this article, we present a novel MROA-DLECGSC technique for disease detection. The presented MROA-DLECGSC method recognizes the presence of heart disease utilizing the ECG signals. To accomplish this, the MROA-DLECGSC technique followed three major processes: data preprocessing, SAETM-based classification, and MROA-based hyperparameter tuning.

### 3.1. Data Preprocessing

In the data preprocessing stage, a group of 3000 ECG signals were utilized for experimental study. While the group of 35 ECG signals contained NULL as classes, it could be removed from the database, and the residual 2965 ECG records could be utilized for simulation. Furthermore, a sampling rate of 100 was chosen from the two sampling rates of 100 and 500 in the database.

### 3.2. ECG Signal Classification Model

For the classification process, the SAETM model was used. The autoencoder (AE) is a DL network to develop an optimum feature description. AEs contain a symmetrical design, and the input and output are the same [18]. Autoencoders contain 3 layers (1 output, 1 input, and 1 hidden layer (HL)). The HL comprises 2 parts: encoded and decoded. The SAE comprises many AEs with a SoftMax layer. An input of the 1st layer of the SAE network is the extraction feature in the EEG signals. The feature can be biased, weighted, and computed with the training of the 1st AE network. This procedure remains for achieving the last abstracted features, and lastly, the result of the final AE network can be utilized for classifying emotions.

**Figure 1:**
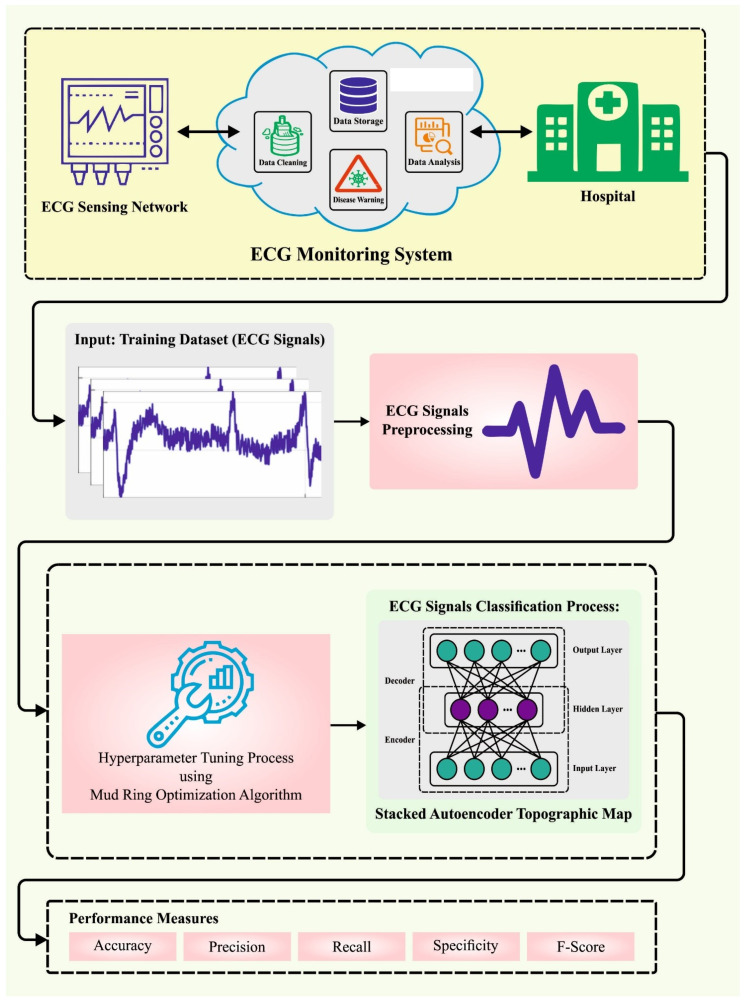
Overall flow of the MROA-DLECGSC algorithm.

During the 1st stage of SAE training, the network utilizes unlabeled data for extracting EEG-abstracted features from the unsupervised model. Afterwards, the encoded part can be accomplished with classification, and it is trained with the supervised method for fine-tuning the SAE parameter. It supports initializing the weighted 1 layer at a time by minimizing reconstruction loss as illustrated in Figure 1.

Let us see the vector of feature extraction in input, and the vector of HL denotes $x\in R^{n}$ and $h\in R^{m}$; correspondingly, n refers to the size of feature extraction from input, and m denotes the size of abstracted features (Equation (1)) (R denotes the real number).

(1) $$h=\sigma \left(x.W+b\right),$$

where $W\in R^{m\times n}$ signifies the weighted matrix, $b\in R^{m}$ denotes the bias vector, and σ implies the activation function (Equation (2)), which is placed from the output layer.

(2) $$\sigma \left(z\right)=\frac{1}{\left(1+e^{-z}\right)}.\;$$

$x\prime \in R^{n}$ stands for the next layer, which is similar in size to the input vectors. The output reconstructs the input vector by upgrading the weighted HL.

(3) $$x^{\prime }=\sigma \left(h.W^{T}+c\right)\;=\sigma \left[\sigma \left(x.W+b\right).W^{T}+c\right].$$

The AE parameters W, $W^{T},$ b, and c are acquired by the back-propagation (BP) technique by square error cost function based on Equation (4) where l is assumed to be the count of trained instances.

(4) $$E=\overset{l}{\underset{k=0}{\sum }}{\left(x-x^{\prime }\right)}^{2}\;$$

The next AE layer was utilized by h, and this process was repeated l times to generate an SAE. An optimal abstracted feature can be created in the HL of all the AE, and $h^{\left(l\right)}$ denotes the optimum representation of abstracted features (Equation (5)).

(5) $$h^{\left(l\right)}=\sigma \left(W^{\left(l\right)}\cdots \sigma \left(W^{\left(2\right)}\sigma \left(W^{\left(1\right)}x+c^{\left(1\right)}\right)+c^{\left(2\right)}\right).+c^{\left(l\right)}\right).\;$$

This particular stage is termed as pretrained to the fixed SAE parameters. An MLP network having 1 resultant neuron can be added to the encoded side of all the SAE for extracting one abstracted feature for plotting the brain map in the topographic map step. $U^{i}$ represents a resultant function in which t denotes the weight matrix, Z refers to the bias vector from the MLP layer, and i stands for the count of SAEs.

(6) $$U^{i}=\sigma \left(\mu h^{\left(l\right)}+\partial \right)i\in \left\{1,2,\;\ldots 32\right\}.$$

where *μ* refers to the reconstruction loss between the input data and the output generated by the SAE. The feature set can be determined as $F^{\left(n\right)}$. It indicates that the features of all the channels can be grouped into 10 parts; the powers are $F_{1},F_{2},F_{3}\;and\;F_{4}$ (4 sub-bands were chosen). The linear EEG features comprising mean, standard deviation, and zero-crossing rate are $F_{5},\;F_{6}$ and $F_{7}$, respectively. Then, $F_{8},\;F_{9}$, and $F_{10}$ were constructed depending on nonlinear features, fractal dimension, approximate entropy, and correlation dimension. The SAE can be built to define the hidden feature abstractions of every channel depending upon Equation (7), where $S_{sae}^{1}\left(x\right),\ldots S_{sae}^{32}\left(x\right)$ signifies the higher feature abstractions of every channel feature.

(7) $$\left\{\begin{array}{l} U^{1}=\sigma \left(\mu h^{\left(l\right)}+\partial \right)=S_{sae}^{1}\left(x\right)\\ U^{2}=\sigma \left(\mu h^{\left(l\right)}+\partial \right)=S_{sae}^{2}\left(x\right)\\ \;\;\;\;\;\;\;\;\;\;\;\;\;\ldots \\ U^{32}=\sigma \left(\mu h^{\left(l\right)}+\partial \right)=S_{sae}^{32}\left(x\right) \end{array}\right.$$

The infrastructure of SAETM can be accomplished by placing 2 neurons from the final layer (Equation (8)), where $y=\left(\begin{array}{c} 1\\ 0 \end{array}\right)$ or $y=\left(\begin{array}{c} 0\\ 1 \end{array}\right)$ depicts the low and high levels of emotion dimensional.

(8) $$y=\sigma \left(\beta U^{i}+\alpha \right).\;$$

where y denotes the resulting function, β indicates the weighted matrix, and α implies the bias vector from the final layer. The fine-tuning step is a vital stage of SAE networks. The fine-tuning system can be utilized for training huge labeled data and enhances classification results. This step fine-tunes the parameter of the final layer of the SAE by the BP technique in the procedure of training with the supervisor.

The layer counts and neuron counts from all the SAE layers are vital in SAETM training. So, the minimal HL and minimal neuron count from all the layers can be vital to have a better classifier. In this case, the Pearson’s or Spearman’s correlation coefficients can be utilized for determining the optimum design. These 2 coefficients compute an optimum similarity among output and input data. These 2 parameters estimate an optimum similarity between input and output data. Thus, the structural loss function (SLF) can be demonstrated in Equation (9).

(9) $$SLF=\omega_{1}\left(1-\rho_{1D_{x}D_{z}}^{2}\right)+\left(1-\omega_{1}\right)\left(1-\rho_{2D_{x}D_{z}}^{2}\right),\;$$

where $\omega_{1}=0.5,$ $\rho_{1D_{x}D_{z}}$, and $\rho_{2D_{x}D_{z}}$ denote the Pearson’s and Spearman’s rank correlation coefficients, respectively, $D_{x}$ denotes the input matrix, and $D_{z}$ shows the resultant matrix.

### 3.3. MROA-Based Hyperparameter Tuning

In this work, MROA was utilized for the optimal hyperparameter selection of the SAETM model. MROA mimics the bottlenose dolphin’s foraging performance, starting with the echolocation-based flooding search and finishing with the development of mud rings to eat [19]. The MRA performance can be introduced to an optimal manner of attaining an objective as a mathematical model. The K parameter becomes an essential optimizer technique as it depicts that the dolphin swarm moves nearby the goal every time the hunting procedure starts. Its parameter adjusts the exchange among the steps of searching for exploitation (mud ring) and prey (exploration) by decreasing the sound volume every time a swarm detects the prey. This section provides a mathematical model of mud ring feeding and seeking food. The stages of MROA are discussed as follows [20]:

**Foraging—Exploration Phase: Echolocation**

This phase includes arbitrarily utilizing dolphins with exploration velocity (V) at locations DE and kinetic energy (K), which could not generate the sound to alert their prey. The assumption is which rate of pulse r’ varies over time and ranges from 0 to 1, where 1 indicates the maximum emission rate and 0 denotes no pulse emission. Dolphins adapt the volume of their sounds based on the vicinity of the potential prey. The computation of vector $K^{\prime }$ can be given as follows:

(10) K=2a×r−a 

(11) $$a=2\left(1-\frac{c_{iier}}{{\mathrm{Max}}_{iter}}\right)$$

A generic solution is used for exploring (hunting prey) in a d-dimension space represented by the proximity K and causes divergence, and finding the optimum prey randomly changes with value >1 or <1. Accordingly, a dolphin selected randomly is preferred instead of the fittest dolphin. This model enables a comprehensive search for the optimal solution. The principles to update the location and velocity should be given. Based on the velocity VE t at a t time step, the workability $D\left(t\right)$ is given as:

(12) $$D\left(t\right)=D\left(t-1\right)+V$$

In Equation (12), a V random vector was utilized as its primary state. The random velocity in the interval [*V*min, *V*max] was first allocated to all the dolphins based on the magnitude of the targeted issue.

**Mud Ring Feed—Phase: Exploitation**

After detection, the dolphin locates and encircles the potential prey. The MROA exploits the optimal or closest prey as the present optimum solution; meanwhile, the optimum design location in the search space is unknown. The better search agent was chosen, and the others consequently changed their location:

(13) $$A=\left|C\cdot D\left(t-1\right)-D\left(t-1\right)\right|\;\;$$

(14) $$D\left(t\right)=D\left(t-1\right)\cdot \;\sin\;\left(2\Pi l\right)-K\cdot A$$

Here, DE denotes the location vector with a better solution globally at $t^{th}$ iteration, with coefficient vectors CE and KE. All dolphins quickly wiggle their tails periodically, like the sign for creating a plume, while the others encircle the target. The vector CE can be computed as follows:

(15) C=2⋅r  

Some locations in the search space might be obtained by finding out random vectors. The prey encircled in this way allows dolphins to defend their position nearby their current best location. The population of random solutions is the MROA searching method initiates (position of dolphin). The dolphin defends the place concerning the optimum location, or a dolphin is selected randomly at every time step. Accordingly, a parameter is reliant on the transition period between exploitation and exploration.

The MROA approach derives a fitness function (FF) for accomplishing improved classifier results. It defines a positive integer defining the enhanced efficacy of candidate solutions. In this work, the minimization of classification error can be considered as the FF, as given in Equation (16).

(16) $$\mathrm{fitness}\left(x_{i}\right)=\mathrm{Classifier}\mathrm{Error}\mathrm{Rate}\left(x_{i}\right)\;=\frac{\mathrm{number}\;\mathrm{of}\;\mathrm{misclassified}\;\mathrm{samples}}{\mathrm{Total}\;\mathrm{number}\;\mathrm{of}\;\mathrm{samples}}\times 100$$

## 4. Results and Discussion

The proposed model was simulated using the Python 3.6.5 tool on PC i5-8600k, GeForce 1050Ti 4GB, 16GB RAM, 250GB SSD, and 1TB HDD. The parameter settings are given as follows: learning rate: 0.01, dropout: 0.5, batch size: 5, epoch count: 50, and activation: ReLU. The performance validation of the MROA-DLECGSC technique was tested on the PTB-XL dataset (https://physionet.org/content/ptb-xl/1.0.3/, accessed on 12 February 2023). The dataset holds five classes: Conduction Disturbance (CD), Hypertrophy (HYP), Myocardial Infarction (MI), ST/T Change (STTC), and Normal ECG (NORM). The classification results were examined in terms of different measures, such as sensitivity ($sens_{y}$), specificity ($spec_{y}$), accuracy ($accu_{y}$), F-score ($F_{score}$), and precision ($prec_{n}$).

**Figure 2:**
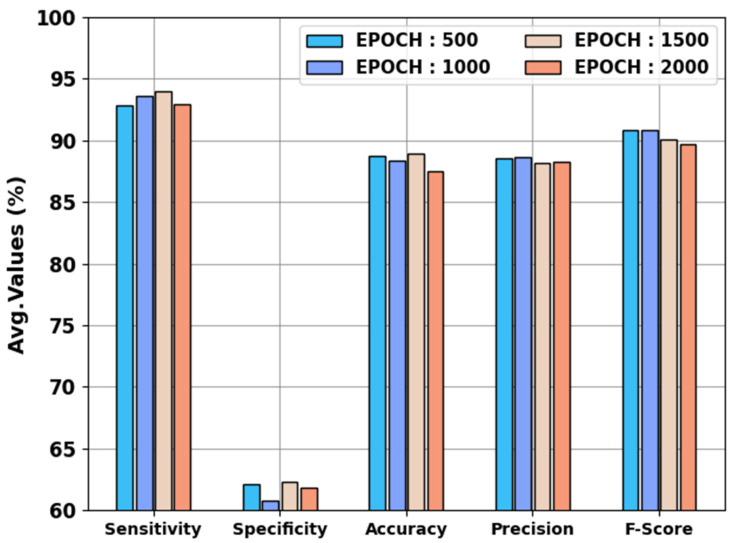
Average outcomes of the MROA-DLECGSC approach under varying epochs.

In Table 1 and Figure 2, the overall ECG classification outcomes of the MROA-DLECGSC technique are investigated under several epochs. The outcomes identify that the MROA-DLECGSC algorithm gains effectual identification of classes under all epochs. For instance, on 500 epochs, the MROA-DLECGSC methodology offers average $sens_{y}$, $spec_{y}$, $accu_{y}$, $prec_{n}$, and $F_{score}$ of 92.83%, 62.12%, 88.75%, 88.59%, and 90.82%, respectively. Concurrently, on 1000 epochs, the MROA-DLECGSC method offers average $sens_{y}$, $spec_{y}$, $accu_{y}$, $prec_{n}$, and $F_{score}$ of 93.65%, 60.77%, 88.32%, 88.68%, and 90.88%, respectively. Simultaneously, on 1500 epochs, the MROA-DLECGSC system offers average $sens_{y}$, $spec_{y}$, $accu_{y}$, $prec_{n}$, and $F_{score}$ of 93.94%, 62.33%, 88.97%, 88.18%, and 90.11%, respectively. Finally, on 2000 epochs, the MROA-DLECGSC approach offers average $sens_{y}$, $spec_{y}$, $accu_{y}$, $prec_{n}$, and $F_{score}$ of 92.94%, 61.86%, 87.50%, 88.27%, and 89.73%, respectively.

**Figure 3:**
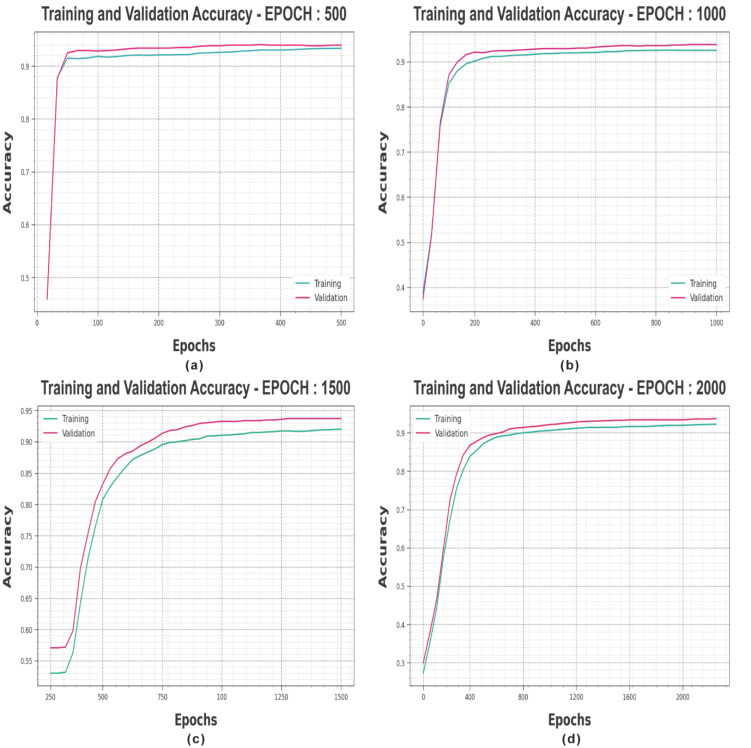
Accuracy curves of MROA-DLECGSC approach (**a**) Epoch: 500, (**b**) Epoch: 1000, (**c**) Epoch: 1500, and (**d**) Epoch: 2000.

Figure 3 shows the accuracy of the MROA-DLECGSC algorithm during the training and validation procedures under varying epochs. The figure indicates that the MROA-DLECGSC technique reaches maximum accuracy values over increasing epochs. In addition, the enhancing validation accuracy over training accuracy depicts that the MROA-DLECGSC algorithm learns efficiently under varying epochs.

**Figure 4:**
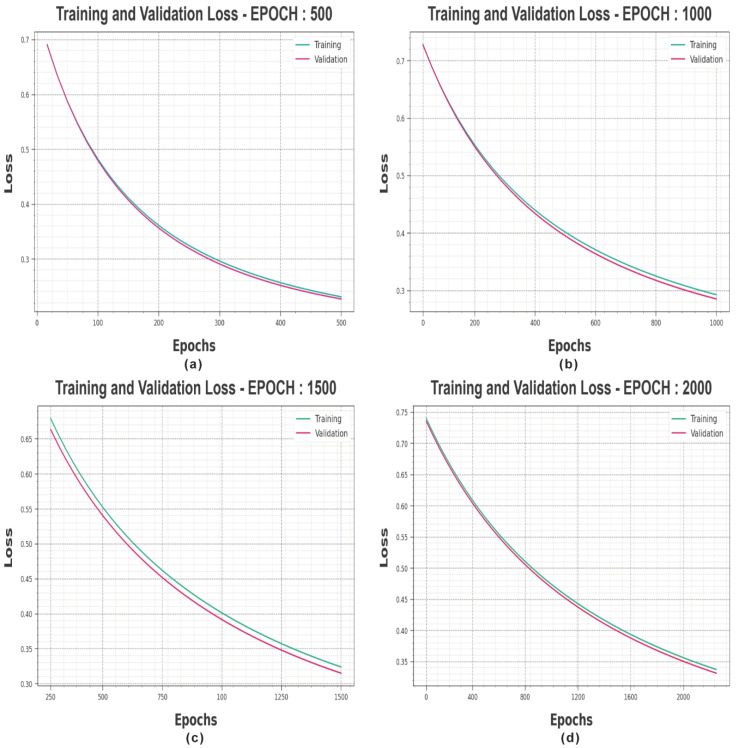
Loss curves of MROA-DLECGSC approach (**a**) Epoch: 500, (**b**) Epoch: 1000, (**c**) Epoch: 1500, and (**d**) Epoch: 2000.

The loss investigation of the MROA-DLECGSC system at the time of training and validation is illustrated under varying epochs in Figure 4. The outcomes imply that the MROA-DLECGSC method obtains closer values of training and validation losses. It can be experimental that the MROA-DLECGSC algorithm learns efficiently under varying epochs.

**Figure 5:**
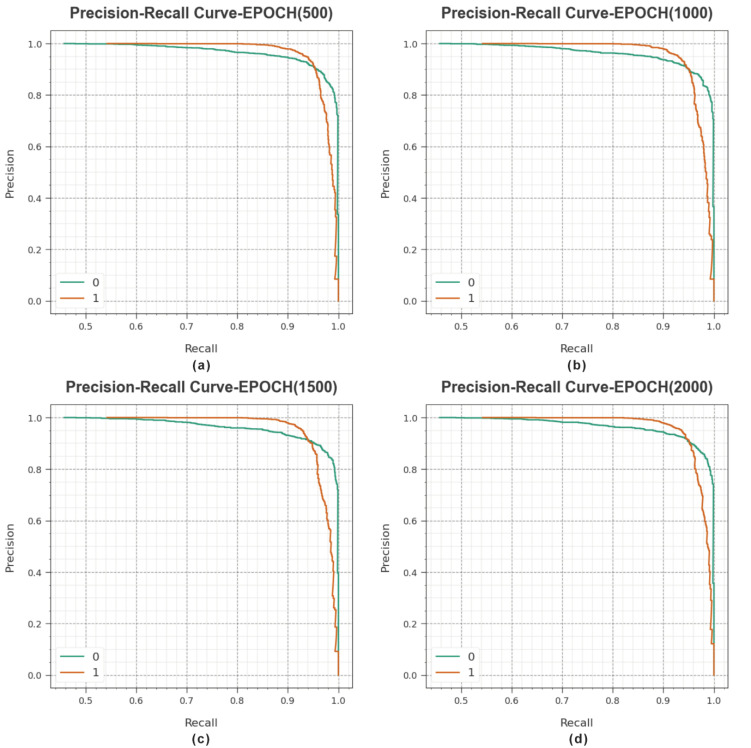
PR curves of MROA-DLECGSC approach (**a**) Epoch: 500, (**b**) Epoch: 1000, (**c**) Epoch: 1500, and (**d**) Epoch: 2000.

A detailed precision–recall (PR) curve of the MROA-DLECGSC algorithm is depicted under varying epochs in Figure 5. The outcomes indicate MROA-DLECGSC algorithm outcomes in maximal values of PR. Furthermore, the MROA-DLECGSC system can gain superior PR values in every class.

**Figure 6:**
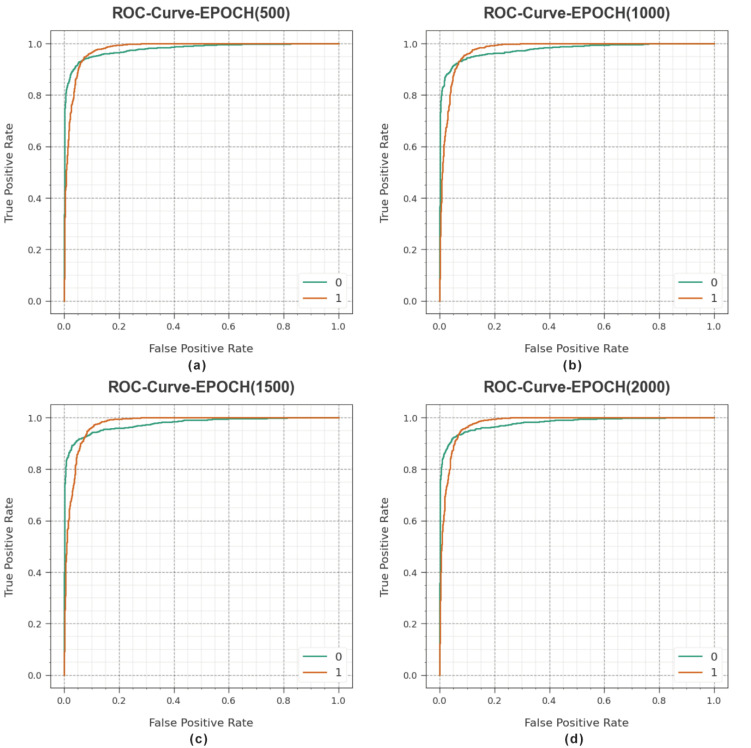
ROC curves of MROA-DLECGSC approach (**a**) Epoch: 500, (**b**) Epoch: 1000, (**c**) Epoch: 1500, and (**d**) Epoch: 2000.

In Figure 6, an ROC analysis of the MROA-DLECGSC method is exposed under varying epochs. The figure states that the MROA-DLECGSC system results in higher ROC values. Moreover, the MROA-DLECGSC algorithm can extend increased ROC values in all classes.

In Table 2, there is a detailed $accu_{y}$ examination of the MROA-DLECGSC methodology with recent techniques [5,21], such as deep learning-based 1D biomedical ECG signal recognition for cardiovascular disease diagnosis (DLECG-CVD), DL-based ECG signal analysis (DL-ECGA), gradient-boosting tree (GBT), random forest (RF), one-dimensional deep convolutional neural network (1-DCNN), logistic regression (LR), decision tree (DT), and K Neighbors Classifier (KNC). The simulation values indicate that the LR and DT models obtained lower $accu_{y}$ of 37.38% and 27.90%, respectively. Then, the RF, 1-DCNN, and KNC models accomplished slightly enhanced $accu_{y}$ of 79.83%, 73%, and 66.89%, respectively. Meanwhile, the DLECG-CVD, DL-ECGA, and GBT models provided closer $accu_{y}$ of 88.24%, 84.70%, and 84.98%, respectively. Nevertheless, the MROA-DLECGSC technique portrayed outperforming results with a maximum $accu_{y}$ of 88.97%. These outcomes confirm the improved outcome of the MROA-DLECGSC algorithm over other methods in the ECG classification process.

## 5. Conclusions

In this article, we presented a novel MROA-DLECGSC algorithm on ECG signals. The proposed MROA-DLECGSC system recognizes the presence of heart disease utilizing the ECG signals. To accomplish this, the MROA-DLECGSC technique follows three major processes: data preprocessing, SAETM-based classification, and MROA-based hyperparameter tuning. Finally, the MROA was executed as a hyperparameter optimizer, which assisted in achieving an enhanced performance. The experimental results of the MROA-DLECGSC algorithm can be tested on the benchmark ECG signal database, and the outcomes depict the better performance of the MROA-DLECGSC technique compared to other recent methodologies. In future, a hybrid DL classifier will be derived to enhance the detection efficacy of the SAETM technique. In addition, future work can focus on the implementation of the proposed model on the real-time IoT-assisted cloud environment. In future, mobile applications can be developed to execute on smartphones and integrate with ECG-monitoring wearables or patches. The application can collect ECG data from the wearable device and process them on the smartphone itself. The ML models can be used for the mobile environment to recognize ECG signals in real time. The application can display the results to the user and transmit relevant data to healthcare professionals for remote analysis and follow-up.

## Figures and Tables

**Table 1:** ECG classifier outcomes of MROA-DLECGSC algorithm under distinct epochs.

EPOCH: 500					
**Measures**	${\mathrm{Sens}}_{y}$	${\mathrm{Spec}}_{y}$	${\mathrm{Accu}}_{y}$	${\mathrm{Prec}}_{n}$	$F_{\mathrm{Score}}$
CD	98.93	57.95	87.77	90.27	94.56
HYP	98.68	66.17	96.46	96.89	98.01
MI	97.68	51.04	91.54	88.20	94.55
NORM	72.99	82.33	79.41	80.20	73.78
STTC	95.85	53.11	88.56	87.39	93.21
**Average**	**92.83**	**62.12**	**88.75**	**88.59**	**90.82**
**EPOCH: 1000**					
**Measures**	${\mathrm{Sens}}_{y}$	${\mathrm{Spec}}_{y}$	${\mathrm{Accu}}_{y}$	${\mathrm{Prec}}_{n}$	$F_{\mathrm{Score}}$
CD	99.65	58.13	91.08	90.08	95.07
HYP	100.44	60.71	94.59	94.13	98.27
MI	98.89	47.36	89.4	90	94.62
NORM	72.92	84.35	77.89	80.71	74.95
STTC	96.35	53.31	88.65	88.48	91.51
**Average**	**93.65**	**60.77**	**88.32**	**88.68**	**90.88**
**EPOCH: 1500**					
**Measures**	${\mathrm{Sens}}_{y}$	${\mathrm{Spec}}_{y}$	${\mathrm{Accu}}_{y}$	${\mathrm{Prec}}_{n}$	$F_{\mathrm{Score}}$
CD	99.23	58.79	91.47	88.96	93.35
HYP	99.43	61.69	95.38	95.23	96.19
MI	99.77	50.88	91.45	91.08	93.12
NORM	72.99	85.06	78.87	79.08	76.14
STTC	98.31	55.24	87.71	86.57	91.79
**Average**	**93.94**	**62.33**	**88.97**	**88.18**	**90.11**
**EPOCH: 2000**					
**Measures**	${\mathrm{Sens}}_{y}$	${\mathrm{Spec}}_{y}$	${\mathrm{Accu}}_{y}$	${\mathrm{Prec}}_{n}$	$F_{\mathrm{Score}}$
CD	97.77	59.38	88.86	90.51	92.82
HYP	98.71	60.59	94.91	96.12	96.3
MI	99.26	50.42	89.85	89.31	92.62
NORM	72.41	83.33	77.32	78.43	75.13
STTC	96.56	55.58	86.59	87	91.82
**Average**	**92.94**	**61.86**	**87.50**	**88.27**	**89.73**

**Table 2:** $Accu_{y}$ outcomes of the MROA-DLECGSC system with other recent methodologies [5,21].

Methods	Accuracy (%)
MROA-DLECGSC	88.97
DLECG-CVD	88.24
DL-ECGA	84.70
GBT	84.98
RF	79.83
1-DCNN	73.00
LR	37.38
DT	27.90
KNC	66.89

## Data Availability

Data sharing does not apply to this article as no datasets were generated during the current study.
